# In Vitro and In Vivo Evaluation of Epidermal Growth Factor (EGF) Loaded Alginate-Hyaluronic Acid (AlgHA) Microbeads System for Wound Healing

**DOI:** 10.3390/jfb14080403

**Published:** 2023-07-28

**Authors:** Maqsood Ali, Si Hyun Kwak, Je Yeon Byeon, Hwan Jun Choi

**Affiliations:** 1 Department of Regenerative Medicine, College of Medicine, Soonchunhyang University, Cheonan 31538, Republic of Korea; 2 Department of Plastic and Reconstructive Surgery, College of Medicine, Soonchunhyang University, Cheonan 31538, Republic of Korea

**Keywords:** alginate, hyaluronic acid, heparin, epidermal growth factor

## Abstract

The management of skin injuries is one of the most common concerns in medical facilities. Different types of biomaterials with effective wound-healing characteristics have been studied previously. In this study, we used alginate (Alg) and hyaluronic acid (HA) composite (80:20) beads for the sustained release of epidermal growth factor (EGF) delivery. Heparin crosslinked AlgHA beads showed significant loading and entrapment of EGF. Encapsulated beads demonstrated biocompatibility with rat L929 cells and significant migration at the concentration of AlgHAEGF100 and AlgHAEGF150 within 24 h. Both groups significantly improved the expression of Fetal Liver Kinase 1 (FLK-1) along with the Intercellular Adhesion Molecule-1 (ICAM-1) protein in rat bone Mesenchymal stem cells (rbMSCs). In vivo assessment exhibited significant epithelialization and wound closure gaps within 2 weeks. Immunohistochemistry shows markedly significant levels of ICAM-1, FLK-1, and fibronectin (FN) in the AlgHAEGF100 and AlgHAEGF150 groups. Hence, we conclude that the EGF-loaded alginate-hyaluronic acid (AlgHA) bead system can be used to promote wound healing.

## 1. Introduction

Human skin works as a protective organ against infections and other external factors. Skin also protects the internal organs from the external environment. Damaged skin may obstruct normal functioning related to daily life [1]. Skin damage causes the loss of important factors that facilitates the regeneration of the skin. Therefore, a moist and warm environment is needed for skin regeneration. Damaged skin goes through different dynamic processes to recover an injured area. During the process of recovery, different factors are generated to enhance skin regeneration [2]. Skin tissue repair aims to restore integrity and regulate biological functions by involving different types of cells. Different types of growth factors and cytokines are released by different cells, which helps in skin tissue repair [3,4]. In contrast, the scar is also considered an important part of the wound-healing process as it prevents dehydration to avoid infections [3,4].

To promote wound healing, different approaches have been developed in the field of regenerative medicine. Mostly, natural polymers are widely used as a primary material [5]. Alginate (Alg) is a naturally derived polymer [6] that is broadly used in tissue engineering to treat acute and chronic injuries by maintaining wound integrity [7]. Alg is used as a biomaterial because of its biocompatibility, degradation, and antibacterial activity [8,9,10]. Hyaluronic acid (Ha) is one of the important parts of the extracellular matrix (ECM), and HA along with alginate is used for the preparation of hydrogels in biomaterials research. [11]. Ha has the efficiency to induce the wound-healing process [12,13]. Epidermal growth factor (EGF) is one of the growth factors that help in wound healing by inducing cell proliferation [14,15,16,17,18]. As proteins are denatured or inactivated after injection for medical treatments, the protection of EGF is crucial in wound healing [19,20,21]. The slow release of the growth factor essentially depends on the nature of biomaterials and cross-linkers used for the system [22]. The combination of the different polymers to optimize the final system is also one of the factors that affect the release of the growth factor as degradation is dependent on the concentration and components of polymers [23,24]. Prior research demonstrated the effects of controlled release of the basic fibroblast growth factor (bFGF) and vascular endothelial growth factor (VEGF) on wound healing [25,26]. Although, certain growth factors are potential candidates for wound healing. As in our previous study, we optimized the vascular epidermal growth factor for wound healing in a dual polymer bead system. Our study has justified the controlled release of VEGF for wound healing [24]. 

We have studied the same system with significant results regarding VEGF in a punch-induced wound rat model. Therefore, in this study, we aimed for the encapsulation of EGF while using dual polymers Alg and HA crosslinked with heparin in Calcium chloride (CaCl_2_) similar to our previous bead system [24]. The bead system is designed to degrade completely along with the total release of EGF in 5 days. As our previous AlgHA bead system exhibited significant results related to wound healing, we aimed to test the effect of the same system along with the EGF for wound-healing purposes. 

Clinically, frequent dressing changes are one of the major barriers and inconveniences in wound healing. Our novel bead system presented in this study is intended to sustain therapeutic concentrations of EGF at the site of the wound, thus eliminating the necessity for frequent or daily dressing changes. This intervention exhibits potential as a viable option for facilitating wound healing, offering advantages in terms of convenience for both patients and healthcare professionals.

## 2. Material and Methods

### 2.1. Fabrication of Crosslinked AlgHA Composite Beads

The bead system was prepared as previously described. Sodium alginate (2%), hyaluronic acid (2%), EGF growth factor (E5036), and heparin sodium salt from porcine intestinal mucosa were purchased from Daejung company (Busan, Republic of Korea) and Sigma Aldrich (Burlington, MA, USA). Alg (2%) and HA (2%) were prepared in distilled water. Beads were prepared in CaCl_2_ at a ratio of 80:20 by mixing 2% Alg (80 mL) and 2% HA (20 mL) to prepare a 100 mL solution, as mentioned in Table 1. Beads were prepared with 5 IU/mL of Heparin (Hep) using 23-gauge needles. A syringe pump, operating at a flow rate of 10 mL/hour, was employed to deliver the solution dropwise into a 5 M CaCl_2_ solution with continuous gentle stirring. The beads were washed three times with distilled water by putting in a beaker over a magnetic stirrer for 5 min/wash and dried overnight at 37 °C.

### 2.2. Scanning Electron Microscopy (SEM) and Energy-Dispersive X-ray Spectroscopy (EDS)

Scanning electron microscopy was employed to examine the cross-section and outer surface of the AlgHA-Hep beads. The selection of dry beads for energy-dispersive X-ray spectroscopy (EDS) was conducted in a random manner. The beads were incised in order to reveal their cross-sectional area. The specimens were affixed to the platform using adhesive carbon tape, which was subsequently coated with gold particles. Subsequently, the samples were examined using (SEM, JEOL, JSM-6701F, Tokyo, Japan) to perform energy-dispersive X-ray spectroscopy (EDS). The energy-dispersive spectrometer (EDS) detector was utilized in the scanning electron microscope (SEM) to obtain data regarding the elemental composition.

### 2.3. Bead Size Distribution and Fourier Transform Infrared Spectroscopy (FT-IR)

Bead size distribution was determined by measuring 20 random beads, using the software Image J (Version 1.8.0) [27] from visual images. AlgHA-Hep beads were prepared according to the method presented in our previous study. The 4500–500 cm^−1^ wavelength was used to record FTIR data (Nicolet iS10—Smart iTR, Thermo Fisher Scientific, Waltham, MA, USA) using OMNIC software.

### 2.4. Beads Weight Loss

The degradation behavior of the beads was studied in phosphate buffer saline pH 7.4 (PBS). Typically, 0.1 g of the 80:20 dry beads were kept in 15 mL PBS on a shaker in an incubator at 37 °C. The sample degradation was checked for 5 consecutive days. The beads of one-time point were allowed to dry for 37 °C and then weighing was carried out to achieve the actual weight. The dynamic weight loss was calculated using the following formula [28]:

(1) $$\mathrm{Degradation}\;(\%)=\frac{\left(w_{1}-w_{2}\right)\times 100}{w_{i}}$$

w_1_ is the weight loss in PBS and w_2_ is the initial dry weight of the beads.

### 2.5. EGF Release Study 

The release study for EGF was carried out in 30 mL PBS (pH 7.4). Typically, 200 mg of dry beads were used for the assay. Two different types of beads, AlgHA and AlgHAHep, were prepared in CaCl_2_. The release of heparin from beads was investigated through the utilization of toluidine blue, with absorbance measurements taken at a wavelength of 590 nm. The encapsulation of beads was carried out by putting beads into the 30 mL EGF-PBS solution (100 ng/mL) for 48 h at 4 °C. The loading capacity and entrapment efficiency were calculated using the following Equations (2) and (3):

(2) $$\mathrm{Loading}\;\mathrm{capacity}=\frac{\mathrm{Total}\;\mathrm{amount}\;\mathrm{of}\;\mathrm{growth}\;\mathrm{factor}\;\mathrm{GF}-\mathrm{Free}\;\mathrm{amount}\;\mathrm{of}\;\mathrm{GF}}{\mathrm{Beads}\;\mathrm{weight}}\times 100$$

(3) $$\mathrm{Entrapment}\;\mathrm{Efficiency}\%=\frac{\mathrm{Growth}\;\mathrm{factor}\;\mathrm{added}-\mathrm{free}\;\mathrm{unentrapped}\;\mathrm{growth}\;\mathrm{factor}}{\mathrm{Growth}\;\mathrm{factor}\;\mathrm{added}}\times 100$$

Additionally, EGF loading, entrapment, and release were analyzed using the EGF ELISA kit (biotechne, Minneapolis, MI, USA). In addition, two distinct sets of beads, specifically AlgHAHepEGF100 and AlgHAEGF100, were prepared for the purpose of conducting the release study. Heparin was incorporated into the AlgHAHepEGF100 bead formulation, whereas the AlgHAEGF100 beads were formulated without the addition of heparin. The beads were incubated for two days at 4 °C in 30 mL of 100 and 150 ng/mL EGF solutions [29] and kept on the 25 rpm rotator. After the assays, the EGF detection ELISA kit was used to examine the quantity of EGF loaded, entrapped, and cumulatively released after 5 consecutive days of sample collection. 

### 2.6. Biocompatibility

The in vitro biocompatibility of AlgHA beads with different concentrations of EGF was observed using rat L929 cells. L929 cells, with a density of 1 × 104 cells per well, were cultured in 24-well hanging plates (SPL Life Sciences, Pocheon, Republic of Korea) using Dulbecco’s Modified Eagle Medium (DMEM) supplemented with 10% fetal bovine serum (FBS) and 1% penicillin. The cells were incubated at 37 °C with 5% CO_2_ in an incubator. EGF-loaded AlgHAEGF50 (50 ng/mL EGF), AlgHAEGF100 (100 ng/mL EGF), and AlgHAEGF150 (150 ng/mL EGF) beads were added to the hanging plate of the wells. The optical density was recorded after 1, 3, and 5 days for (3-(4,5-Dimethylthiazol-2-yl)-2,5-diphenyltetrazolium bromide) at 595 nm for MTT assay.

Furthermore, an equivalent quantity of L929 cells was subjected to fixation using a 4% solution of paraformaldehyde (PFA) obtained from Sigma Aldrich, located in St. Louis, MO, USA. Permeabilization was achieved by utilizing a 0.5% solution of Triton x-100 (Bio-Rad Laboratories in Redmond, Redmond, WA, USA). A blocking solution consisting of bovine serum albumin (2.5%) was employed. Alexa 488 antibody (Invitrogen, Thermofisher, Carlsbad, CA, USA) was used for staining the cytoskeleton and 1 µg ml^−1^ Hoechst 33342 (Invitrogen, Thermofisher, Carlsbad, CA, USA) was used for staining the nuclei in L929 cells. The stained specimens were observed using a fluorescence microscope with a 40× magnification. (Olympus, FV10i-W, Tokyo, Japan).

### 2.7. In Vitro Wound-Healing Migration Assay

For in vitro cell migration, L929 cells (5 × 10^5^) were cultured in Dulbecco’s Modified Eagle Medium (DMEM) with 10% horse serum and 1% penicillin and allowed to grow in 35 mm plates labeled as control, AlgHAEGF50, AlgHAEGF100, and AlgHAEGF150 in a CO_2_ incubator at 37 °C. After reaching confluency of up to 95%, a straight-line scratch was made using a 10 µL sterile pipette tip. Culture media was treated with different EGF groups and used for the migration studies of L929 cells. Images were taken at 0 and 24 h by an AXIO microscope (Zeiss, Baden-Württemberg, Germany), and L929 cell migration was analyzed by using Image J [27].

### 2.8. Immunoblotting

Rat bone-marrow-derived mesenchymal stem cells (rbMSCs) (2 × 10^3^) were cultured along with AlgHAEGF50, AlgHAEGF100, and AlgHAEGF150 for 14 days. A lysis buffer and protease inhibitor were used for protein extraction to check the expression of certain markers. Following the isolation of a specific quantity of protein utilizing bovine albumin serum (BSA), the protein samples, measuring 40 µg, were subjected to separation via sodium dodecyl sulfate-polyacrylamide gel electrophoresis. Subsequently, the gel was transferred onto the hydrophilic polyvinylidene fluoride (PVDF) membrane. The protein bands were visualized using ultraviolet light following the blocking and staining process with primary and secondary antibodies. Antibodies such as Flk-1 and ICAM-1 were used for optimizing the concentration of EGF for in vivo implantation. Relative quantification of the protein blots was performed by Image J [30]. 

### 2.9. Animal Models and Surgical Procedures

This in vivo study was approved under the SCH22-0021 number by the ethical committee of Soonchunhyang University. Two weeks before the in vivo plan, 18 male rats (8 weeks old) were ordered from Daehan Biolink Co., Ltd. (DBL, Eumseong-gun, Republic of Korea. The rats were kept at a controlled temperature (22–23 °C). Rats were divided randomly, considering 9 rats/week studies (3 in the control, 3 in the AlgHAEGF100 group, and 3 in the AlgHAEGF150 group). Food and water were provided regularly until the sacrifice.

Rats were anesthetized by using isoflurane. The back side of the rat was trimmed, and an 8 mm sterile punch was used to induce wounds by maintaining a distance of 4–5 cm between the two wounds to avoid each other’s effects. Three groups, namely defect only (control), AlgHAEGF100, and AlgHAEGF150, were processed for in vivo transplantation. AlgHAEGF100 and AlgHAEGF150 beads were loaded in sterilized syringes and then implanted into the punch-induced wound. Wounds were covered with a Tegaderm transparent film (3M, St. Paul, MN, USA) after the implantation of 10–12 beads. Rats were sacrificed at 1 week and 2 weeks after the implantation. Postoperative photographs were taken by a Canon EOS 90D (Canons^®^, Tokyo, Japan) Camera from a standard distance of 40 cm to capture defect sites. Skin tissues were fixed in 4% PFA after collection.

### 2.10. Histology

For histological analysis, the PFA-fixed tissues were processed for dehydration by using a graded ethanol solution. Xylene was used as a clearing solution. The final cleared tissues were embedded in melted paraffin. The tissue blocks were then sectioned into 5 and 10 µm sizes for staining. Hematoxylin and eosin (H&E) staining and immunohistochemistry (IHC) were carried out for histological analysis. 

## 3. Statistical Analysis

The experiments were conducted in triplicate. The experimental data are presented in the form of means accompanied by their corresponding standard deviations. Statistical significance among the different samples was assessed using GraphPad Prism 8, employing a one-way analysis of variance (ANOVA) and an unpaired t-test. Statistical significance was determined by *p*-values of <0.01 and <0.001. 

## 4. Results 

### 4.1. Characterization of AlgHA-Heparin Beads 

**Figure 1:**
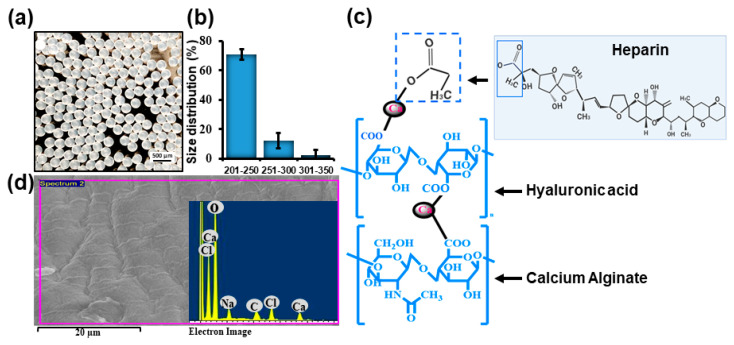
(**a**) Visual photographs of AlgHAHep beads, (**b**) the percentages of bead size distribution, (**c**) illustration of possible AlgHAHepEGF chemical composition, and (**d**) EDS spectrum indicating the composition of the bead system.

**Figure 2:**
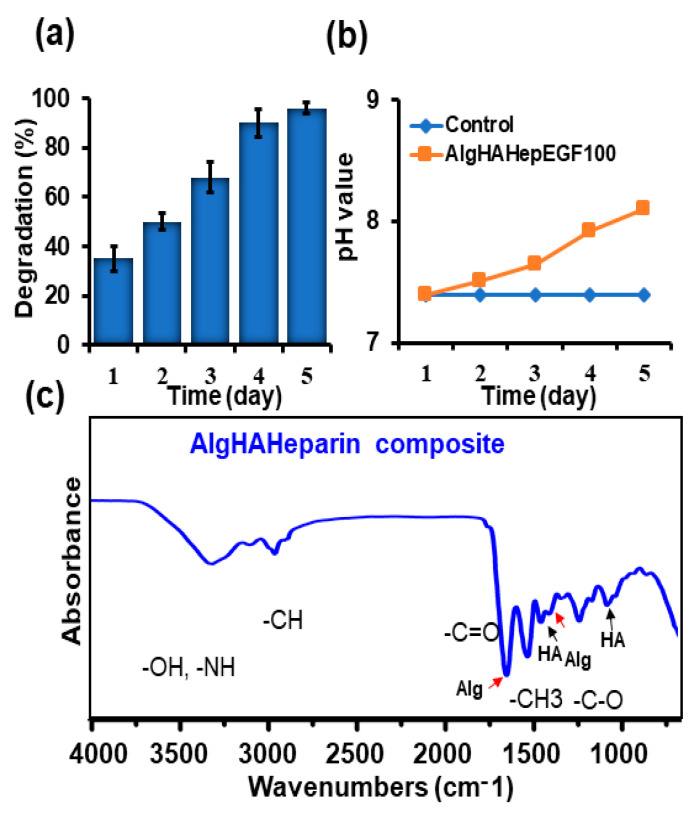
Characterization of prepared AlgHAHepEGF beads, (**a**) degradation of the bead system (n-3), (**b**) pH change of PBS with the AlgHAHepEGF100, and (**c**) the FTIR analysis of the bead system showing the peaks related to alginate, hyaluronic acid, and amide bonds.

AlgHA beads were prepared based on the previously described method [24]. ALG80: HA20 beads were combined with 5 IU heparin. The homogenous beads were prepared as shown in Figure 1a and Figure S1 in the range of 200 to 350 µm Figure 1b. Approximately 70% of beads were 200 to 250 microns in size. The expected chemical composition of the beads was similar to our previous study, as mentioned in Figure 1c. Equal-size beads were prepared based on our previous findings. Figure 1d EDS shows peaks for calcium, chloride, oxygen, etc. The EDS results exhibited similarity with the data of previous studies [31,32]. The beads’ sizes were well distributed according to the difference in size presented in Figure 1b. The selection of AlgHA beads was performed based on degradation. Figure 2a,b show 100% degradation at day 5 and the pH value of AlgHA-HepEGF100, respectively. The beads were kept in a shaking incubator at 37 °C, which might be the reason for the degradation in PBS. Moreover, acidic polymers may undergo gradual neutralization in the PBS, which might lead to a slightly basic pH in the PBS. The optimized beads were processed for EGF release. The FTIR spectroscopy of the AlgHA-Hep composite demonstrated the availability of all the relevant peaks related to Alg and HA in Figure 2c.

### 4.2. EGF Loading, Entrapment, and Cumulative Release

**Figure 3:**
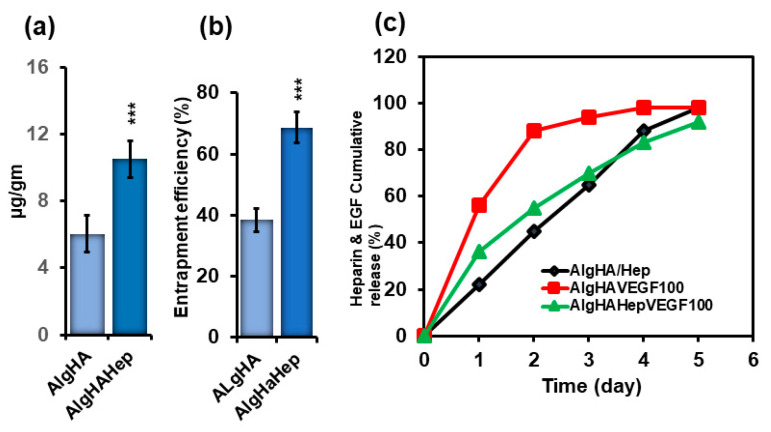
(**a**) EGF loading on beads (AlgHA and AlgHAHep), (**b**) EGF entrapment efficiency (%) of the beads (AlgHA and AlgHAHep), and (**c**) heparin and EGF release from the beads; AlgHA/Hep shows the cumulative release of heparin from the beads and AlgHA100 (EGF loaded with no heparin) and AlgHAHepEGF100 (heparin crosslinked EGF) show the cumulative release of EGF (*n* = 3, *** *p* < 0.001, 200 mg/group).

To evaluate the sustained EGF release, loaded and entrapped EGF in the beads was evaluated before the cumulative release of EGF. EGF was loaded into two groups: AlgHA and AlgHAHep. AlgHA showed approximately 6.0 µg/gram of loaded EGF while the groups with heparin (AlgHAHep) showed a significant amount (10.5 µg/gram) of EGF loaded as shown in Figure 3a. AlgHAHep entrapped approximately 70% EGF compared to AlgHA (Figure 3b), thereby demonstrating the significance of heparin as a cross-linker for the controlled release of EGF. Figure 3c shows the cumulative release of EGF. Briefly, two groups were preceded for EGF release: AlgHAEGF100 and AlgHAHepEGF100. In Figure 3c, AlgHAEGF100 shows the delayed cumulative release of EGF due to heparin. The AlgHAEGF100 bead system showed a slow cumulative release of EGF while the bead system without heparin showed a burst release of EGF of approximately 85% within 2 days. AlgHAHepEGF100 showed a controlled release of EGF, releasing approximately 50% of the loaded EGF within 48 h. The results show the efficiency of heparin bonding with a number of different proteins [33] and cross-linking capabilities for the controlled release studies as mentioned in previous studies [24,34].

### 4.3. MTT Assay and Proliferation

**Figure 4:**
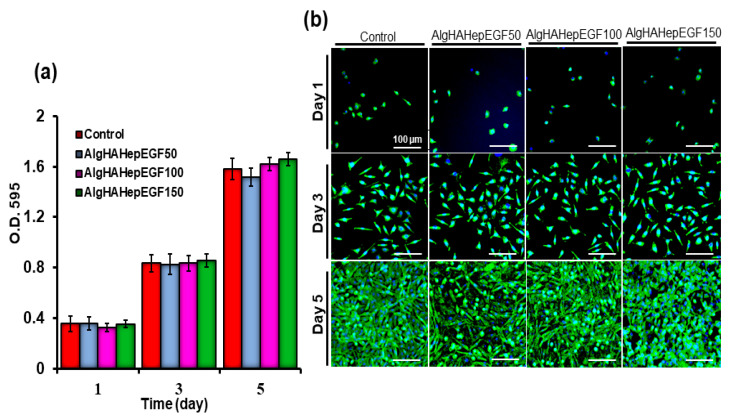
Biocompatibility testing of L929 with the AlgHA scaffold groups by MTT (**a**) after 1, 3, and 5 days of treatment and (**b**) nucleus fluorescence microscopic analysis of the L929 cells for cell proliferation by Hoechst staining (Scale bar 500 µm).

For biocompatibility with the L929 fibroblasts, AlgHAEGF50-, AlgHAEGF100-, and AlgHAEGF150-treated media were preceded for MTT assay. L929 fibroblasts were chosen for biocompatibility because prior research demonstrated that EGF increases the production of VEGF and hepatocyte growth factor (HGF) by fibroblasts, which promote cell proliferation [35]. Alg and HA composites along with the control were used previously for checking their biocompatibility (Figure S2) [24]. The L929 cells showed growth promotion on days 3 and 5. Our results demonstrate the compatibility of AlgHAEGF groups with L929 cells at different time points as shown in the bar graph in Figure 4a. Figure 4b shows fluorescence microscopic images of cell proliferation on days 1, 3, and 5. Our results are in line with previous studies on the proliferation of L929 [36,37]. The L929 culture showed an increase in the confluency of the plate with time. Almost the total area of the plate showed confluency after a 5-day cell culture as also demonstrated in previous studies [6]. The AlgHA bead system along with EGF is considered a suitable system for in vivo studies based on their non-toxic behavior towards L929 cells.

### 4.4. Effect of EGF on the Migration of L929 Cells 

**Figure 5:**
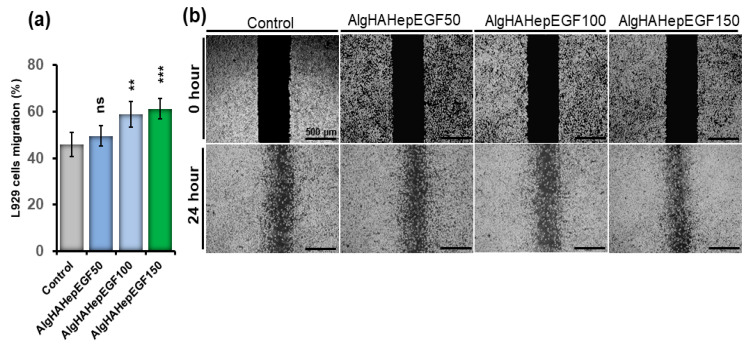
Representation of cell migration (**a**) control group along with the EGF group’s effect on L929 after 24 h and (**b**) representative images of L929 cell migration (*n* = 3, ns: non-significant, ** *p* < 0.005, *** *p* < 0.001).

L929 cells were employed to check the efficiency of EGF on cell migration. Different concentrations of EGF were used to study the migration of L929 cells. Furthermore, different concentrations of EGF were employed to check their effect on L929 cell migration. We observed that all the groups showed cell migration as can be seen in Figure 5a. L929 cells showed migration in the AlgHAEGF50, AlgHAEGF100, and AlgHAEGF bead systems in the presence of 150 ng/mL of EGF (Figure 5b). AlgHAEGF100 and AlgHAEGF150 ng/mL showed significant cell migration after 24 h. These findings positively attest to the previous cell migration results [38,39,40,41].

### 4.5. Western Blot Analysis

**Figure 6:**
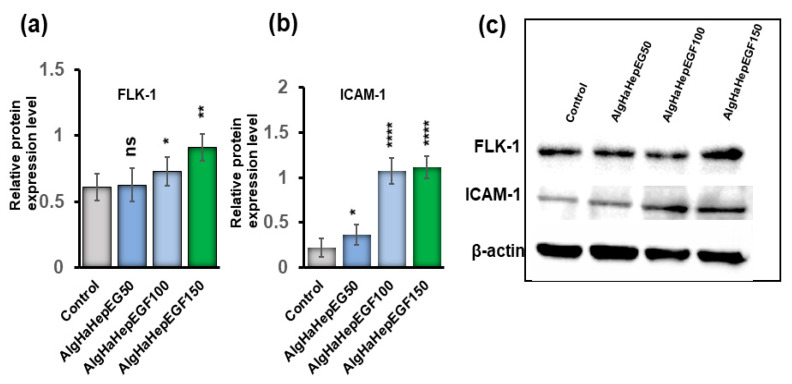
Representative Western blots graph and images. (**a**) Flk-1 protein expression obtained by three independent experiments, (**b**) ICAM-1 expression, and (**c**) representative blot images of Flk-1, ICAM-1, and beta-actin expressions in the control and different AlgHAHepEGF groups (*n* = 3, ns: non-significant, * *p* < 0.005, ** *p* < 0.001, **** *p* < 0.0001).

The bead system was tested for the expression of certain markers that can promote epidermal growth. The groups AlgHAEGF50, AlgHAEGF100, and AlgHAEGF150 were analyzed for the expression of epidermal markers in rbMSCs on days 7 and 14. EGF plays a significant role in neo-epidermal formation by stimulating Flk-1 [42,43] while the ICAM-1 signaling pathway has demonstrated regulatory behavior by EGF in previous studies [44,45]. Therefore, FLK1 and ICAM-1 antibodies were used for rbMSCs differentiation. Day 7 showed slight non-significant relative expressions of Flk-1 and ICAM-1 as shown in Figure S3. Figure 6a,b shows the relative expression of Flk-1 and ICAM-1 proteins on day 14. Figure 6c shows the representative blots. All the EGF groups showed significant expression of Flk-1 and ICAM-1. As AlgHAEGF100 and AlgHAEGF150 groups showed markedly significant expressions of Flk-1 and ICAM-1, the two groups were considered for in vivo implantation. 

### 4.6. Wound Closure

**Figure 7:**
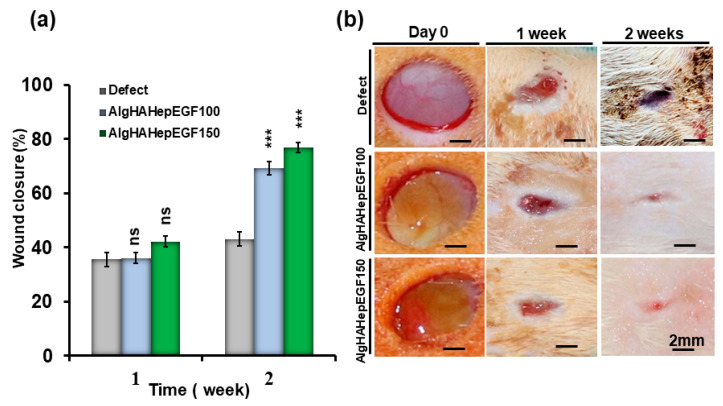
(**a**) Wound-healing behavior of the control and AlgHA groups is shown with relative comparison and statistics of 1 week and 2 weeks after wound closure. (**b**) Representative photos of the wound sites in the three groups at 1 and 2 weeks (*n* = 3, ns: non-significant, *** *p* < 0.001).

After the implantation of the bead system for wound healing, all the rats were checked for any kind of blood leakage and no bleeding was found. To investigate rat skin wound closure by AlgHAEGF100 and AlgHAEGF150, wounds at days 0, 7, and 14 were analyzed. Figure 7a shows all the wound closure percentages. On day 7, all the groups showed approximately 40% closure of the wound. At day 14, significant wound closure was observed compared to the control group. AlgHAEGF100 and AlgHAEGF150 showed approximately 69% and 77% wound area closure, respectively. Figure 7b shows wound images of the different groups at different time points along with the scale bars. Both EGF treatment groups showed significant potency for wound closure. 

### 4.7. Post-Implantation Histological Analysis

**Figure 8:**
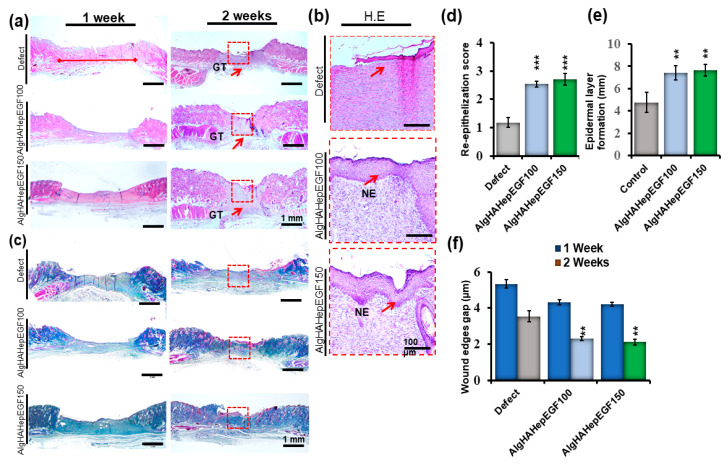
Histological findings showing that AlgHAHepEGF150 promotes wound healing. (**a**) 1 week and 2 weeks after inducing the wound, defect sites show contraction in 2-week images (scale bar 1 mm), (**b**) magnified images of the defect and AlgHA groups showing epithelial thickness and formation of the epidermal layer across the wound area (scale bar 100 µm), magnified images of the defect and treatment groups showing vascularization, (**c**) MT staining of all the groups at 1 and 2 weeks; images show collagen formation and granulation tissue formation in all the groups (scale bar 1 mm), (**d**) epithelial score between the two ends of defect and treatment groups, (**e**) epidermal layer formation graph of all the 2 weeks groups, and (**f**) wound closure gap of the two edges of the defect in AlgHAHepEGF100 and AlgHAHepEGF150 groups. NE (neo-epithelization) *n* = 3, unpaired *t*-test analysis, ** *p* < 0.01, *** *p* < 0.001) (Red squares shows wound sites while arrows pointing new epidermal layer in the wound area).

To further investigate the effect of the bead system on the internal repair of the skin tissue and regeneration, the rat punch wound model was used after days 7 and 14 and analyzed via H &E (Figure 8a,b) and Masson trichrome (MT) staining (Figure 8c) [46]. Figure 8a shows the efficiency of the beads system for wound healing. The gap between the two edges of the wound was confirmed by H&E staining. The day 7 wound showed mild closure in all the groups. Day 14 edges showed gap filling between them as quantified in Figure 8f. Day 14’s results showed mild granulation, while in AlgHAEGF100 and AlgHAEGF150, increased formation of new granulation tissue along with inflammatory cells was observed as shown in Figure 8a,b. The EGF effect shows the thickness of the epidermal line in day 14 groups. Figure 7b shows the epithelial thickness and epidermal layer formation. The defect areas showed the significant formation of the new epidermal layer in AlgHAEGF100 and AlgHAEGF150 groups as shown in Figure 8d while Figure 8e shows contraction between the two edges of the wound with certain epidermal layer growth. AlgHAEGF100 and AlgHAEGF150 showed significant epidermal layer formation as compared to the control group. M.T. staining showed collagen formation in all the groups. The defect model exhibited mild new collagen formation while AlgHAEGF100 and AlgHAEGF150 treatment groups demonstrated an increase in the formation of new collagen formation as shown in Figure 8c. The healed areas showed the formation of endothelial cells forming vessels and certain gland-like structures. EGF groups in our system facilitate the thickness and formation of the epidermal layer, vessels, and glandular structures, and our results are in line with Jiasheng Xu et al. regarding EGF [47]. 

### 4.8. Immunohistochemistry

**Figure 9:**
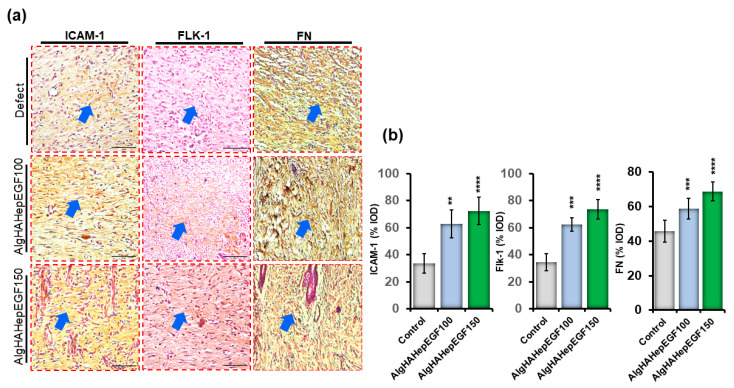
Immunohistochemistry for ICAM-1, Flk-1, and fibronectin in rat skin. (**a**) Microscopic images of ICAM-1, Flk-1, and FN for defect, AlgHAHepEGF100, and AlgHAHepEGF150 groups and (**b**) % integral optical density (% IOD) of ICAM-1, Flk-1, and FN (*n* = 3, ** *p* < 0.005, *** *p* < 0.001, **** *p* < 0.0001) (Blue arrows identifying stained areas in the image).

In the current study, ICAM-1, Flk-1, and FN staining was observed after 2 weeks of implantation (Figure 9a). The treatment groups (AlgHAEGF100 and AlgHAEGF150) showed significant expression of all the mentioned expression proteins (Figure 9b) after 2 weeks of implantation. The ICAM-1 protein is another important marker that is expressed in the process of wound healing, and the absence of ICAM-1 has been reported to delay wound healing [48]. EGF stimulates Flk-1 neo-epidermal formation in previous studies. Figure 9b shows the elevated expression of ICAM-1 in the AlgHAEGF100 and AlgHAEGF150 groups as compared to the control group. It is hypothesized that ICAM-1 and Fibronectin (FN) expressions in the treatment group may lead to wound healing internally and external closure by promoting angiogenesis [49]. Figure 9b shows the significant expression of ICAM-1 and FN in the AlgHAEGF100 and AlgHAEGF150 groups. The expression of all the above-mentioned antibodies in the rat skin demonstrates the efficiency and importance of the EGF-loaded AlgHA bead system in wound healing. 

## 5. Discussion

Wound healing is a complicated step-by-step process of cellular and biochemical reactions. These involve inflammation, cell migration, proliferation, and remodelling [50]. Alg and HA are used widely in regenerative medicine [5]. Both polymers are non-toxic and have the capability of cell delivery and clinical applications [51,52,53]. Previously, we optimized AlgHA beads by a combination of different ratios of alginate and hyaluronic acid along with the vascular endothelial growth factor (VEGF) for wound-healing purposes [24]. In this study, we synthesized AlgHA beads with sizes ranging from 200 to 350 µm with the combination of specific alginate and hyaluronic acid ratios. The bead system shows complete degradation after 5 days and slightly elevated pH values. We implanted almost the same size of the beads in order to achieve the same degradation time for all the beads. The complete degradation of the beads might be allowing the formation of new tissue [54]. The AlgHAEGF100 showed a slight increase in pH due to the degradation of beads and release of the growth factor. The FTIR results demonstrated bonding between the elements in the system, which is in line with our previously presented FTIR data [24]. Two groups, AlgHA (without heparin) and AlgHAHep (cross-linked with heparin), were prepared for evaluating the efficiency of heparin. Heparin loading does not affect AlgHAHep bead size, although one study mentioned that nanoparticles might show swelling after cross-linking with heparin [55]. Heparin is one of the macromolecules and possesses a negative charge [56]. Heparin is a suitable cross-linker for different growth factors used in different biomaterials [57]. Heparin is a highly charged molecule with consistent units of negatively charged sulfated glycosaminoglycan (GAG) chains that can interact with different growth factors and biomaterials to facilitate the slow release of certain growth factors to facilitate wound healing [58,59,60]. In addition, heparin plays a significant role in the immune defenses of the human body. The substance exhibits antioxidant, anti-inflammatory, and vasodilator characteristics [61]. Heparin exerts an influence on the hemostatic phase of wound healing through its interaction with different molecules [62]. Additionally, heparin functions as a powerful anti-inflammatory substance by suppressing the activity of enzymes and cytotoxic mediators that are released by pro-inflammatory cells [62]. There are also some potential negative effects of heparin observed in different studies on wound healing; these include induced thrombocytopenia [63], bleeding complications [64], delayed wound closure [65], and interactions with other medications [66]. 

EGF was significantly loaded and entrapped in the AlgHAHep beads as compared to AlgHA. Heparin is an excellent cross-linker for certain growth factors that facilitates the controlled release of heparin [67,68]. AlgHAHep100 shows the slow release of the EGF in 5 days as compared to the AlgHA group. Our study shows more than 70% uniform bead sizes as mentioned in Figure 1b, which might be due to the homogenous mixture of heparin in the AlgHA solution as one study shows the possible effect of heparin on nanoparticle sizes [55]. The EGF release pattern shows almost 100% of the loaded EGF in 5 days. Heparin cross-linked beads show the slow release of EGF. Days 1, 2, 3, 4, and 5 show 36%, 55%, 70%, 83%, and 92% cumulative release of EGF, respectively, while beads without heparin show fast cumulative EGF release starting from 56% at day 1 and 88% at day 2. The slow release of EGF facilitates wound healing in our rat models over time. Recombinant heparin-binding-EGF showed wound healing in a previous study, and researchers showed significant reepithelization in murine models [69]. 

In our study, the AlgHA group showed excellent biocompatibility with the L929 cells. The cells showed elevated values at day 3 and 5 time points. AlgHAEGF100 and AlgHAEGF150 shows slightly elevated optical density (O.D.) values at day 5, which confirm the role of active EGF in cell growth and migration. EGF also facilitates cell migration, and our results show significant L929 cell migration after 24 h. Our results conform with the findings of previous studies [70]. Two groups (AlgHAEGF100 and AlgHAEGF150) showed highly significant expressions of Flk-1 and ICAM-1 in rbMSCs. EGF stimulates Flk-1, which is one of the VEGF receptors that might stimulate neo-epidermal formation after inducing angiogenesis [42,43]. The ICAM-1 signaling pathway has demonstrated regulatory behavior in previous studies [44] and revealed enhancement in ICAM-1 expression [45]. Therefore, we chose two groups for in vivo implantation in rat models.

Wound healing goes through proliferation and remodeling after the initial stages of hemostasis and inflammation. Previously, EGF has shown efficiency in facilitating wound closure phenomena in 14 days [71]. In our study, in vivo implantation of two treatment groups shows significant wound closure within 2 weeks. AlgHAEGF100 and AlgHAEGF150 groups showed non-significant wound closure at the 1-week time point. Several molecular mechanisms are involved in wound healing. EGF binds to EGF receptors on target cells that can initiate intracellular signaling REF. EGFR activation leads to the mitogen-activated protein kinase (MAPK) pathway and phosphatidylinositol 3-kinase (PI3K)/Akt pathways [72]. These pathways play a role in regulating cell migration, differentiation, survival, and proliferation [73]. The promotion of cell proliferation is facilitated by the activation of the MAPK pathway through the action of EGF. The pathway that has been activated induces the upregulation of genes that are associated with the progression of the cell cycle, resulting in an enhanced rate of cell proliferation at the site of the wound. This process facilitates cellular proliferation, thereby facilitating wound healing [74].

EGF has potency for epidermal layer formation [75], and in the current study, the histological examinations showed the effect of EGF groups on wound healing. Our study showed the formation of granulation and a new epidermal layer along with some unclosed areas. Our results are in line with the study related to the effect of EGF on wound healing [76]. The study’s findings on wound closure confirm a reduction in the distance between the borders of the wound in histological analysis. IHC staining was performed to identify the progressive markers for the wound-healing process in the presence of the epidermal growth factor. The receptor tyrosine kinase (Flk-1) marker is known as one of the earliest genetic markers to be expressed during the process of wound healing. Flk-1 and ICAM-1 are known to play a crucial role in the development of cardiac endothelial cells, embryonic vasculature, and several pro-inflammatory cytokines, respectively [77,78,79]. Another factor, fibronectin (FN), enhances the wound-healing effect by utilizing stem cells [49] and promoting angiogenesis [80]. Our significant IHC findings positively attest to the previously mentioned studies regarding Flk-1, ICAM-1, and FN expressions. 

Overall, continued exposure of the EGF in the wound area promotes tissue repair. The cell receptors have a high affinity for binding with the EGF [81,82], but 10 to 12 h of exposure is required for DNA synthesis. Previous data demonstrated that the EGF receptors at the cell surface decreased until the amount of EGF become low. Increased and constant occupancy of the receptors was observed after 6 h [83,84]. Therefore, our bead system is focused on the controlled release of the EGF for prolonged availability to achieve wound healing. 

Some of the limitations of the current study should be taken into consideration. Rats are commonly utilized as an animal model in the field of wound-healing research. Nevertheless, it is important to recognize the potential differences in the wound-healing mechanisms between rats and humans. Therefore, it is important to acknowledge that the direct applicability of findings derived from studies conducted on rats to the human population may not always be easy to understand. Rats have a significant capacity for wound contraction, a characteristic that holds the potential to impact the interpretation of research outcomes. The reliability of using wound contraction observed in rats as a representation of wound healing mechanisms in humans is questionable, given that wound closure in humans is primarily dependent on re-epithelialization and collagen deposition. Moreover, differences in wound size and the healing rate between rats and humans may exist. The rate at which rat skin wounds heal is comparatively faster than that of human wounds, which may have implications for evaluating therapeutic interventions and enhancing the understanding of wound repair mechanisms.

## 6. Conclusions

In this study, an AlgHA bead system was formulated to achieve the sustained release of EGF and promote wound closure and healing. AlgHAEGF groups showed migration and biocompatibility with L929 fibroblasts in in vitro studies. The treatment groups also demonstrated enhanced wound closure and increased epidermal layer formation along with endothelial vascularization in the wounds. Immunohistochemistry analysis of the treatment groups showed significant expressions of ICAM-1, Flk-1, and FN. Our innovative bead system is intended to keep therapeutic amounts of EGF at the wound site, eliminating the need for daily or frequent dressing changes. It may be a promising candidate for wound healing by providing convenience to both patients and healthcare providers.

## Figures and Tables

**Table 1:** Preparation of AlgHA beads.

Beads Type	Alginate (2%) (*w/v*)	Hyaluronic Acid (2%) (*w/v*)	Heparin
AlgHAHep	80 mL	20 mL	5 IU/ml
AlgHA	80 ml	20 ml	No heparin added

## Data Availability

Not applicable.

## Supplementary Materials

The following supporting information can be downloaded at: https://www.mdpi.com/article/10.3390/jfb14080403/s1. Figure S1, Visual appearance of AlgHa-heparin beads. Scale bar of 500 µm. Figure S2, L929 fibroblasts (control) and AlgHA-heparin beads’ MTT results. Figure S3, Day 7 FLK-1 and ICAM-1 expression in RBMSCs (a and b).
