# Supplementary material for: In Vitro and In Vivo Evaluation of Epidermal Growth Factor (EGF) Loaded Alginate-Hyaluronic Acid (AlgHA) Microbeads System for Wound Healing
Source: J Funct Biomater. 2023 Jul 28;14(8):403. doi: 10.3390/jfb14080403 (PMC10455903; doi:10.3390/jfb14080403)

Figure S1: Visual appearance of AlgHa-heparin beads. Scale bar of 500 μm.

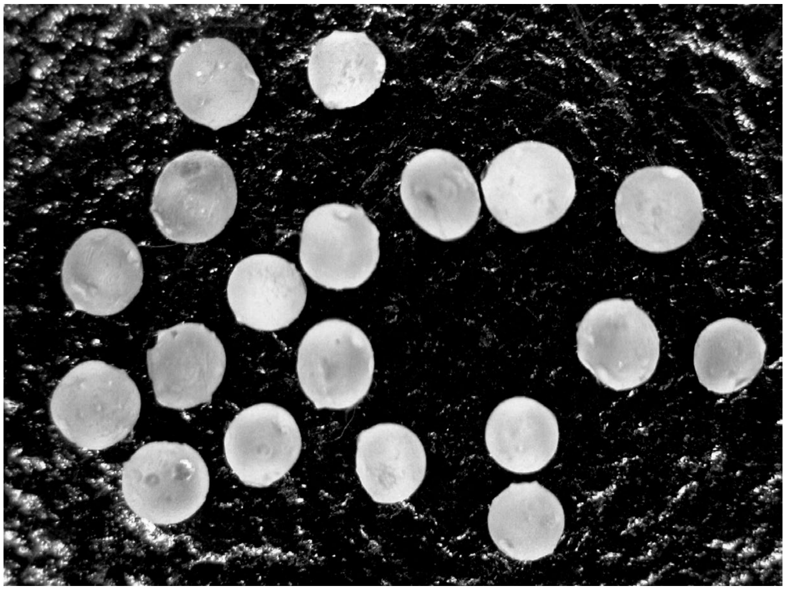

Figure S2: L929 fibroblasts (control) and AlgHA-heparin beads' MTT results.

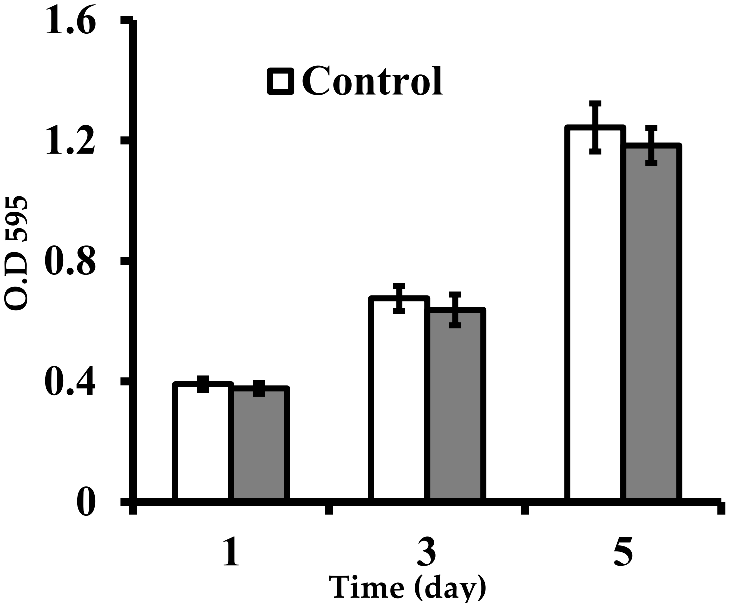

Figure S3: Day 7 FLK-1 and ICAM-1 expression in RBMSCs (a and b).

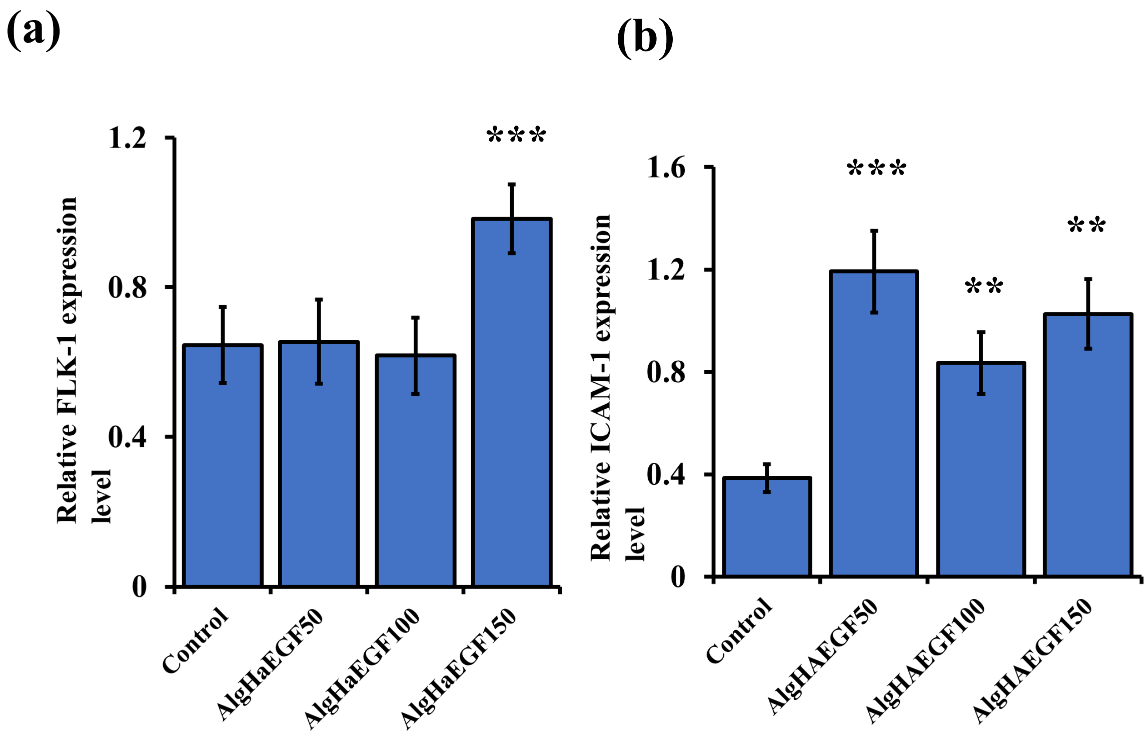

Supplement: Supplementary file 1 [file jfb-14-00403-s001.zip › jfb-2490127-supplementary.pdf]
